# Diagnostic Accuracy of Biomarkers of Alcohol Use in Patients With Liver Disease: A Systematic Review

**DOI:** 10.1111/acer.14512

**Published:** 2020-12-25

**Authors:** Janique Arnts, Benedict T. K. Vanlerberghe, Sylvia Roozen, Cleo L. Crunelle, Ad A. M. Masclee, Steven W. M. Olde‐Damink, Ron M. A. Heeren, Alexander van Nuijs, Hugo Neels, Frederik Nevens, Jef Verbeek

**Affiliations:** ^1^ From the Division of Gastroenterology and Hepatology (JA, BTKV, AAMM) Department of Internal Medicine Maastricht University Medical Center Maastricht The Netherlands; ^2^ Governor Kremers Centre‐Maastricht University Medical Centre (SR) Maastricht The Netherlands; ^3^ Department of Psychiatry (CLC) Vrije Universiteit Brussel (VUB) Universitair Ziekenhuis Brussel (UZ Brussel) Brussels Belgium; ^4^ Toxicological Center (CLC, AN, HN) University of Antwerp Antwerp Belgium; ^5^ NUTRIM School of Nutrition and Translational Research in Metabolism (AAMM, SWMO‐D) Maastricht University Maastricht The Netherlands; ^6^ Department of Surgery (Maastricht University Medical Center Maastricht The Netherlands; ^7^ Department of General, Visceral and Transplantation Surgery (RWTH University Hospital Aachen Aachen Germany; ^8^ Division of Imaging Mass Spectrometry (RMAH) Maastricht MultiModal Molecular Imaging (M4I) Institute Maastricht University Maastricht The Netherlands; ^9^ Department of Gastroenterology and Hepatology (FN, JV) University Hospitals KU Leuven Leuven Belgium

**Keywords:** Diagnostic Accuracy, Alcohol Use Biomarkers, Liver Disease

## Abstract

**Background and Aims:**

Alcohol‐related liver disease is the most frequent cause of cirrhosis and a major indication for liver transplantation. Several alcohol use biomarkers have been developed in recent years and are already in use in several centers. However, in patients with liver disease their diagnostic performance might be influenced by altered biomarker formation by hepatic damage, altered excretion by kidney dysfunction and diuretics use, and altered deposition in hair and nails. We systematically reviewed studies on the diagnostic accuracy of biomarkers of alcohol use in patients with liver disease and performed a detailed study quality assessment.

**Methods:**

A structured search in PubMed/Medline/Embase databases was performed for relevant studies, published until April 28, 2019. The risk of bias and applicability concerns was assessed according to the adapted quality assessment of diagnostic accuracy studies‐2 (QUADAS‐2) checklist.

**Results:**

Twelve out of 6,449 studies met inclusion criteria. Urinary ethyl glucuronide and urinary ethyl sulfate showed high sensitivity (70 to 89 and 73 to 82%, respectively) and specificity (93 to 99 and 86 to 89%, respectively) for assessing any amount of alcohol use in the past days. Serum carbohydrate‐deficient transferrin showed low sensitivity but higher specificity (40 to 79 and 57 to 99%, respectively) to detect excessive alcohol use in the past weeks. Whole blood phosphatidylethanol showed high sensitivity and specificity (73 to 100 and 90 to 96%, respectively) to detect any amount of alcohol use in the previous weeks. Scalp hair ethyl glucuronide showed high sensitivity (85 to 100%) and specificity (97 to 100%) for detecting chronic excessive alcohol use in the past 3 to 6 months. Main limitations of the current evidence are the lack of an absolute gold standard to assess alcohol use, heterogeneous study populations, and the paucity of studies.

**Conclusions:**

Urinary and scalp hair ethyl glucuronide are currently the most validated alcohol use biomarkers in patients with liver disease with good diagnostic accuracies. Phosphatidylethanol is a highly promising alcohol use biomarker, but so far less validated in liver patients. Alcohol use biomarkers can complement each other regarding diagnostic time window. More validation studies on alcohol use biomarkers in patients with liver disease are needed.

## Graphical abstract

Alcohol use biomarkers can be of great benefit, but concerns exist regarding their applicability in patients with liver disease. Arnts, Vanlerberghe et al. provide an in‐depth review of their diagnostic accuracy in liver patients. Urinary and scalp hair ethyl glucuronide are the most validated alcohol use biomarkers. The diagnostic accuracy of phoshatidylethanol is highly promising, but so far less validated in liver patients. Their review aids in the correct application and interpretation of these alcohol use biomarkers in daily practice.

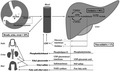

Alcohol‐related liver disease (ALD) is the most frequent cause of cirrhosis and the most frequent indication for liver transplantation (LTx; EASL, 2018). Objective and accurate markers to assess alcohol use can have a major impact on the care for patients with ALD (EASL, 2018). Alcohol use biomarkers need to have a high diagnostic accuracy. Biomarkers with low sensitivity may label patients with active alcohol use as abstainers and biomarkers with low specificity may label abstainers as active alcohol users. In patients not adhering to alcohol abstinence, psychosocial and psychiatric support can be intensified to achieve abstinence (Khan et al., 2016), which might prevent (further) hepatic and extra‐hepatic damage. Alcohol use biomarkers can also play a role in the selection process for LTx. In most centers, a period of abstinence is required to be eligible for LTx (EASL, 2018). Currently, the assessment of alcohol abstinence and ongoing alcohol use remains a major diagnostic challenge. Physicians mainly have to rely on information provided by the patient and their family. This information might be unreliable because of fear of stigmatization and fear that the transplant team will delay or disallow LTx if recent or active alcohol use becomes known (Schieber et al., 2015). Equally as important, accurate alcohol use biomarkers can also confirm alcohol abstinence in LTx candidates and prevent false accusation. Furthermore, these biomarkers can play a role in the post‐LTx setting by enabling early detection of alcohol relapse and in clinical trials in ALD patients (Wurst et al., 2015). Alcohol use biomarkers also can have an important role in the legal setting, for example, in decision making at the court on reobtaining or maintaining a license for drivers or operators of heavy machinery after alcohol‐related incidents (Palmer, 2009). Courts can also use them to assess abstinence in child custody or visitation disputes (Palmer, 2009). In addition, postmortem investigations of alcohol intake can give valuable information on the cause of death of the person both in clinical and forensic setting (Palmer, 2009).

Direct measurement of alcohol in blood, exhaled breath, or urine is considered as the gold standard (EASL, 2018). However, these methods only detect alcohol ingested in the last hours because of its rapid elimination. Routinely applied indirect markers like mean corpuscular volume (MCV) and the liver tests gamma glutamyl transferase (GGT), aspartate aminotransferase (AST), and alanine aminotransferase (ALT) lack diagnostic accuracy as alcohol biomarkers, especially in the presence of liver disease (Gough et al., 2015). Among hepatologists and other healthcare workers, the indirect biomarker carbohydrate deficient transferrin (CDT) is widely used. Serum CDT has a half‐life of circa 15 days and can detect repeated excessive alcohol use of more than 50 g/d for more than 1 to 2 weeks (Crunelle et al., 2016; EASL, 2018). CDT can detect excessive alcohol use up to 4 weeks before analysis (Crunelle et al., 2016). Alcohol and its metabolites inhibit glycosyltransferases and induce sialidases resulting in formation of CDT (Fig. 1), but its applicability in patients with liver disease is controversial (Arndt, 2001).

**Fig. 1:**
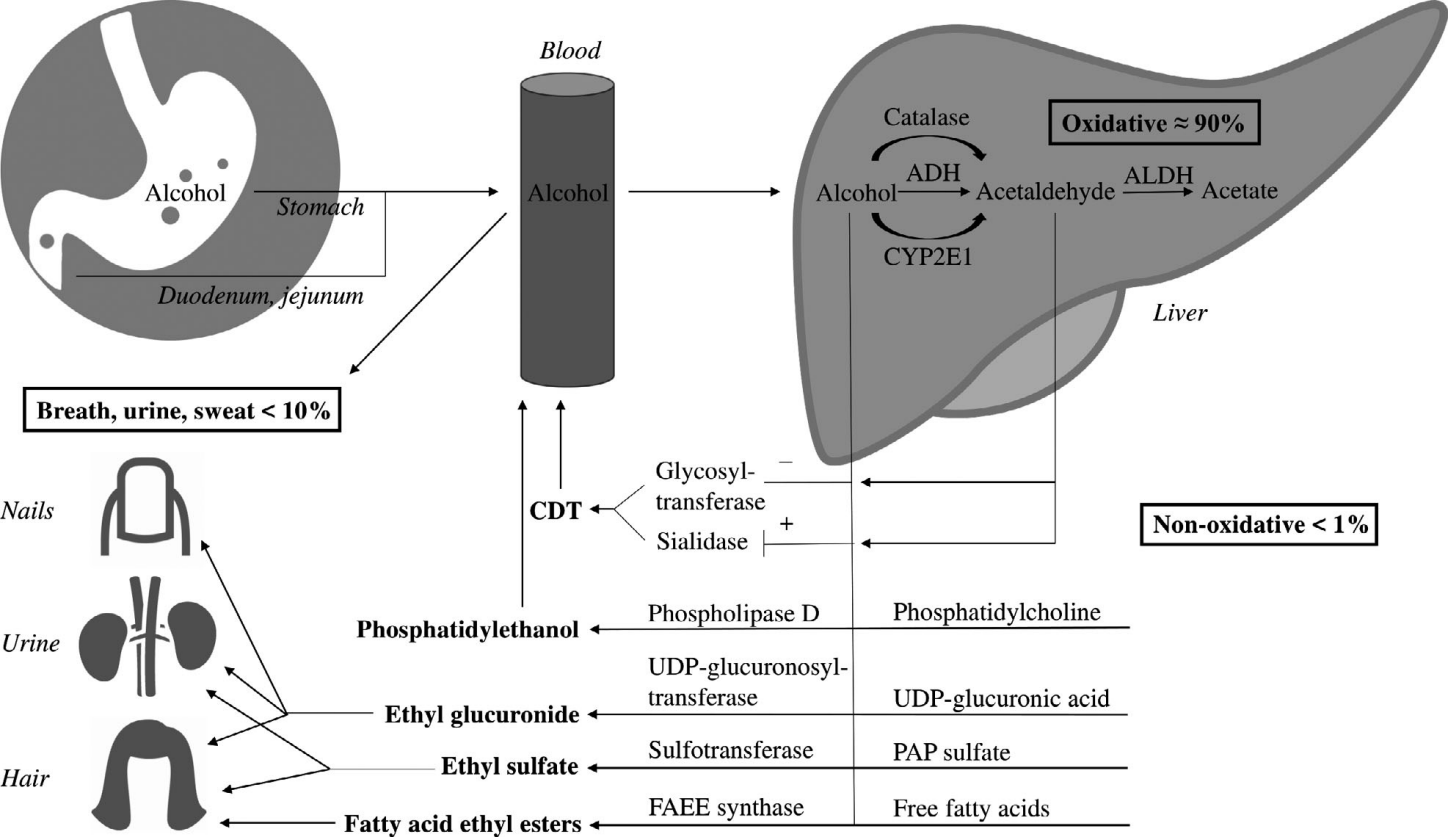
Schematic overview of the oxidative and nonoxidative alcohol metabolism. ADH, alcohol dehydrogenase; ALDH, acetaldehyde dehydrogenase; CYP2E1, cytochrome P450 2E1; FAEE, fatty acid ethyl ester; PAP sulfate, 3′‐phosphoadenosine‐5′‐phosphosulfate; UDP‐glucuronic acid, uridine 5′‐diphosphoglucuronic acid; UDP‐glucuronosyltransferase, uridine 5′‐diphosphoglucuronosyltransferase (UGT).

In the last years, increasing attention has been given to direct alcohol markers (i.e., nonoxidative alcohol metabolites) because of their potential higher diagnostic accuracy (EASL, 2018). However, these markers were primarily tested in patients without liver disease. In particular, ethyl glucuronide (EtG) gained attention. EtG is the product of glucuronidation of alcohol catalyzed by uridine 5′‐diphosphoglucuronosyltransferases (UGTs) and is formed in hepatocytes, the gastro‐intestinal tract and the kidney (Fig. 1; Heier et al., 2016). EtG accumulates in hair and nails (Cappelle et al., 2017). Hair EtG (hEtG) reflects the alcohol consumption in the past months, depending on the analyzed hair length taking into account the growth rate of hair of approximately 1 cm/month (Crunelle et al., 2014; Pragst and Balikova, 2006). Nails grow significantly slower than hair, resulting in higher absolute concentration of EtG in nails than in hair (Fosen et al., 2017). Fosen and colleagues investigated the elimination kinetics of EtG in nail clippings in forty patients in alcohol rehabilitation. The half‐life of nail EtG was 13.3 days (range: 5.5 to 29) and patients (*n* = 22) who reached negative nail EtG during the study period did that after 47.0 days (range: 13–60; Fosen et al., 2017).

EtG can also be detected in urine (uEtG) after ingestion of already small amounts of alcohol (i.e., <10 g) where it remains present for up to several days after intake (EASL, 2018; Heier et al., 2016). In alcohol‐dependent patients with an initial alcohol concentration of >1 g/l, uEtG (cutoff = 0.5mg/l LC‐MS) can be detected up to 130 hours (range: 40 to 130 hours [median 78]) after initial testing (Helander et al., 2009) and similar results can be found in intoxicated healthy patients (Borucki et al., 2005). Detection times of uEtG correlated weakly with initial alcohol concentration in the above‐mentioned study (Helander et al., 2009). In addition, urinary ethyl sulfate (uEtS), the product of enzymatic sulfonation of alcohol by sulfotransferases (SULTs) in the liver, intestine, and lung (Fig. 1), has been described as an accurate direct biomarker for the assessment of recent alcohol use (EASL, 2018; Heier et al., 2016). UEtS has similar elimination kinetics as uEtG (Helander et al., 2009).

Another direct and promising biomarker that assesses alcohol consumption in the prior weeks is phosphatidylethanol (PEth) tested in whole blood or dried blood spots (Heier et al., 2016; Varga et al., 2000). PEth is an alcohol‐derived phospholipid formed from phosphatidylcholine, mainly in red blood cell membranes, by a transphosphatidylation reaction catalyzed by phospholipase D in the presence of alcohol (Fig. 1; Heier et al., 2016; Helander and Zheng, 2009). PEth can detect a single drinking event over the last 3 to 12 days and the half‐life of PEth following a single drinking event (Blood alcohol content [BAC] = 1 g/l) after 2 weeks of abstinence is 3 days (Schröck et al., 2017). After longer periods of drinking (5 subsequent days with BAC = 1 g/l after 3 weeks of abstinence), its half‐life is approximately 4 to 10 days in healthy subjects (Gnann et al., 2012). Patients with chronic alcohol abuse might have on average shorter half‐life of PEth (4 days; Varga et al., 2000), but heavily drinking subjects can have a positive PEth after 5 to 6 weeks of abstinence (Stewart et al., 2014).

The application of 1 or more of these alcohol use biomarkers is gaining interest and is already part of the routine work‐up for LTx in some centers. (EASL) Therefore, their diagnostic accuracies are extremely important because of the potential impact of a negative or positive test result on clinical decision making. Liver disease might impair the diagnostic performance of aforementioned alcohol biomarkers due to impaired biomarker formation in damaged hepatocytes or altered renal biomarker elimination by the presence of kidney dysfunction or use of diuretics (Cederbaum, 2012; Fig. 2). In addition, slower hair growth in cirrhotic patients might impair the sensitivity and specificity of biomarkers tested in hair (EASL, 2018; Fig. 2). Therefore, in this study, we systematically reviewed studies on the diagnostic accuracy of biomarkers of alcohol use in patients with liver disease and performed a detailed quality assessment of these studies.

**Fig. 2:**
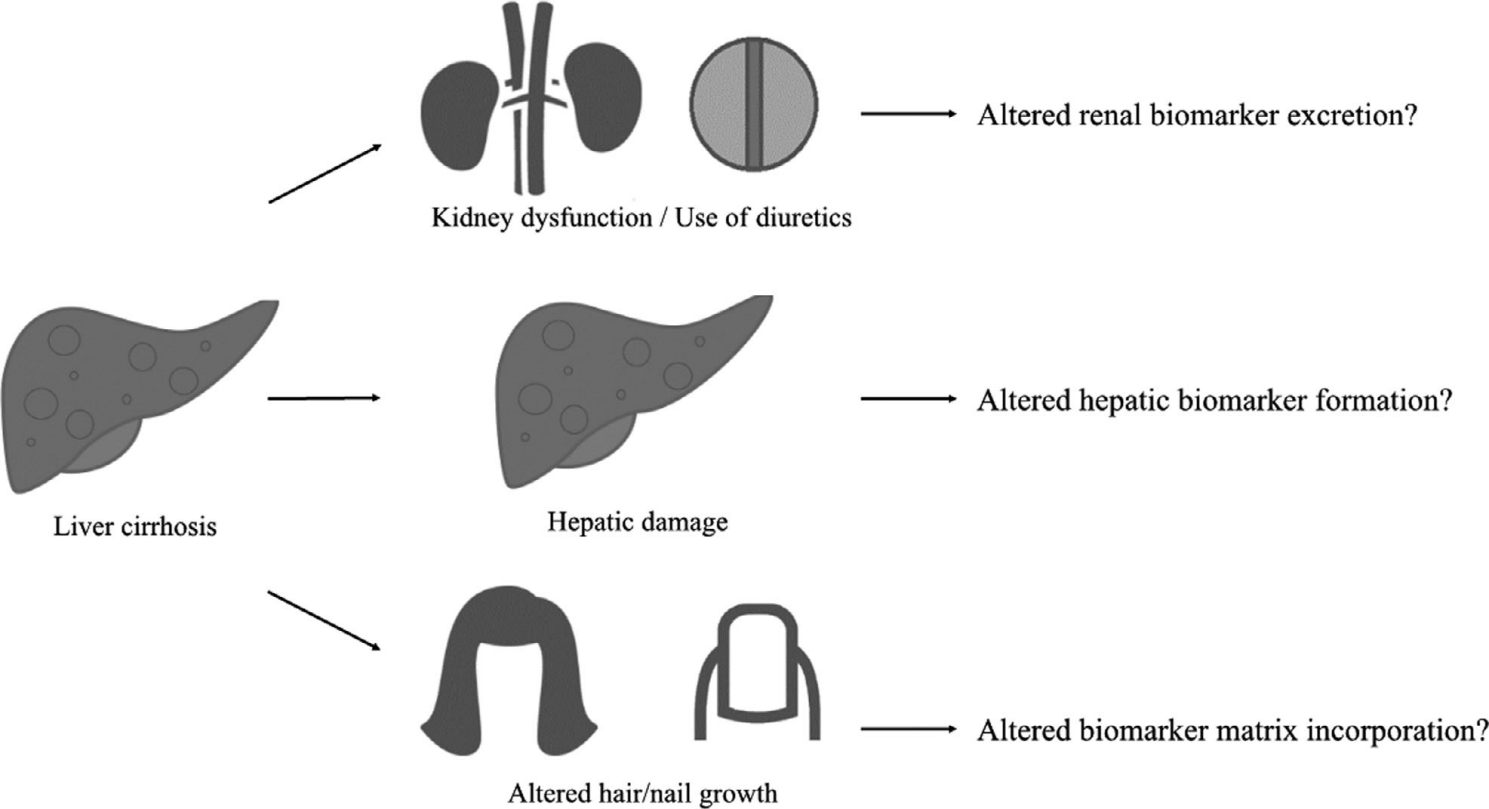
Possible theoretical methods impairing the diagnostic accuracy of alcohol use biomarkers in patients with liver disease.

## Materials and Methods

Reporting of this systematic review was performed using the preferred reporting items for systematic reviews and meta‐analyses (PRISMA) guidelines (Liberati et al., 2009).

### Search

A structured search in PubMed/Medline/Embase databases was performed for relevant studies, published from 1960 until April 28, 2019. The selected keywords and synonyms searched in titles and abstracts were as follows: ((((cirrhosis OR hepatitis OR liver) AND (alcohol* OR ethanol OR ethyl) AND (biomarker OR marker OR detect* OR monitor* OR CDT OR EtG OR EtS OR FAEE OR phosphatidylethanol) AND English [Language] NOT (review OR pregnan* OR animal OR mouse OR mice OR rat OR rats)))). In addition, references of selected articles were assessed and included if suitable.

### Eligibility Criteria

Studies were included when meeting the following inclusion criteria: (i) Studies were original research articles written in English and published in peer‐reviewed journals; (ii) studies assessed the diagnostic accuracy of direct and/or indirect biomarkers for a defined quantity or range of alcohol use or alcohol abstinence; (iii) the reported diagnostic accuracy of the biomarker contained sensitivity (SE), specificity (SP), and optionally positive predictive value (PPV) and negative predictive value (NPV); (iv) the study population or a subgroup of the study population consisted of patients with liver disease; (v) liver disease was specified according to its etiology and severity (at least cirrhosis vs. no cirrhosis); and (vi) up‐to‐date methods for CDT and glycoprotein analysis were used, that is, immunonephelometry (N‐latex), capillary zone electrophoresis (CZE), or high‐performance liquid chromatography (HPLC) in case of CDT (Wielders et al., 2017).

The exclusion criteria included the following: (i) review articles, commentaries, letters to the editor, editorials; (ii) animal studies, studies in pregnant women and postmortem studies, because of possible differences in biomarker physiology and kinetics compared to the study population of interest, that is, patients with liver disease; (iii) studies on blood‐, breath‐, or urine alcohol levels; (iv) studies validating alcohol questionnaires; (v) studies in which the only markers of interest were standard blood analyses such as MCV or (isoenzymes or ratios of) the serum liver tests, that is, GGT, AST, ALT, AST/ALT ratio, and mitochondrial AST; and (vi) studies using outdated methods of CDT‐ or glycoprotein analysis, that is, isoelectric focusing (IEF), small column ion exchange chromatography (CDTect), radioimmunoassay (RIA), enzyme immunoassay (EIA), or turbidimetric immunoassay (TIA; Bortolotti et al., 2018; Hagan et al., 2014).

### Study Selection

**Fig. 3:**
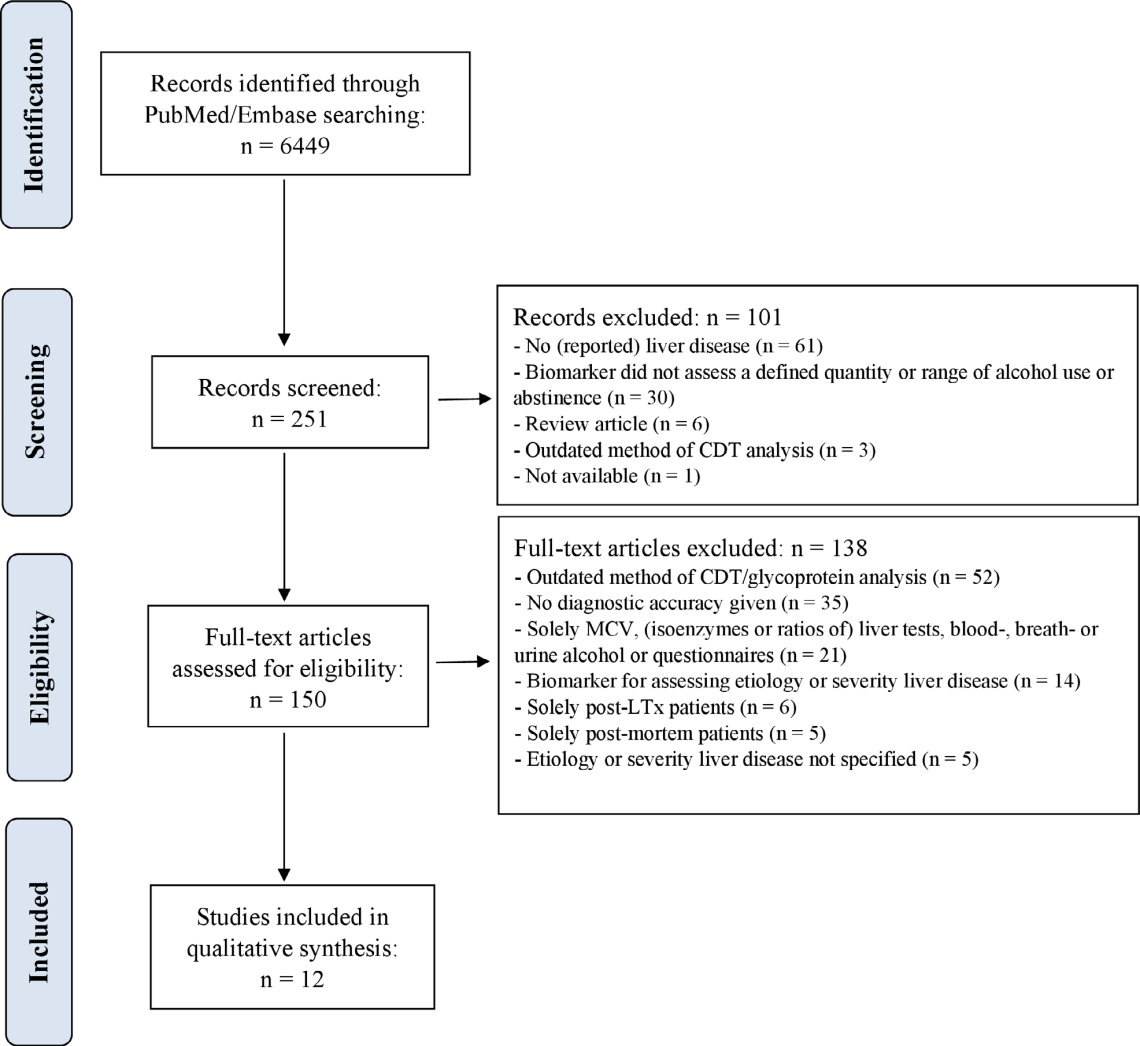
Flowchart demonstrating study identification and selection. CDT, carbohydrate deficient transferrin; LTx, liver transplantation; MCV, mean corpuscular volume.

A flowchart of the study selection process is presented in Fig. 3. A total of 150 articles were eligible for full text review, after which 138 extra articles were excluded. In 52 studies, an outdated method for CDT/glycoprotein analysis was used. In 35 studies, diagnostic accuracy of the alcohol biomarker was not reported. These studies did not report sensitivity and/or specificity or only reported a statistical correlation of the alcohol biomarker with the amount of alcohol intake or a statistical difference in biomarker concentration between different drinking groups. In 5 studies, liver disease severity was not specified. These studies did not report the presence or rate of patients with cirrhosis. Screening of references of the included studies revealed no relevant missing studies. Method and results of the study quality assessment using the PRISMA guidelines and the adapted quality assessment of diagnostic accuracy studies‐2 (QUADAS‐2) can be found in the Supplement 1: Quality of included studies. Included articles were independently assessed for quality by 2 of the researchers (J.A and S.R.). Performing a meta‐analysis was not considered to be appropriate because of the highly heterogeneous study populations, study designs, and the small number of eligible studies per alcohol use biomarker. Overall, 12 studies met the eligibility criteria and were included in this systematic review.

## Results

### Study Characteristics and Reported Outcomes

We reported the included studies according to their theoretical diagnostic time window, that is, short‐term (uEtG and uEtS), mid‐term (CDT and PEth), and long‐term (hEtG; Tables 1, 2, 3).

**Table 1:** Diagnostic Accuracy of Short‐Term Biomarkers of Alcohol Use in Patients With Liver Disease

Study	Diagnostic time window	Study population	Reference standard	Method	Cutoff value	Diagnostic accuracy			
						SE	SP	PPV	NPV
*Biomarker: ETG in urine*									
Staufer and colleagues (2011)	NS	Total: *n* = 141 LTC with alcoholic liver cirrhosis: *n* = 105 LTR with history of alcoholic liver cirrhosis: *n* = 36 Alcohol use total group: NS	Self‐report EtOH MeOH CDT	EIA + LC‐MS/MS	≥500 ng/ml	LTC + LTR, any use			
						89.3	98.9	89.3	98.9
					≥1,000 ng/ml	LTC + LTR, any use			
						75	99.3	91.3	97.5
Stewart and colleagues (2013)	3–7 days	Total: *n* = 120 HCV: *n* = 41; ALD: *n* = 25; HCV + ALD: *n* = 13; NAFLD: *n* = 16; other: *n* = 25 Of which cirrhosis: *n* = 65, median MELD 10 Alcohol use total group: median 16 drinks/wk	Self‐report Clinical assessment CDT PEth	LC‐MS/MS	>100 ng/ml	Cirrhotics + noncirrhotics, 3 days, any use			
						76	93	81	91
						Cirrhotics + noncirrhotics, 7 days, any use			
						70	99	97	85
Piano and colleagues (2014)	NS	Total: *n* = 121 LTC with alcoholic liver cirrhosis: *n* = 98 LTR with history of alcoholic liver cirrhosis: *n* = 23 Alcohol use total group: mean 14.1 drinks/wk	Self‐report EtOH (blood, urine) CDT	EIA	>500 ng/ml	LTC + LTR, any use			
						89.2	98.8	97.1	95.4
Andresen‐Streichert and colleagues (2017)	7 days	Total: *n* = 112 LTC with alcoholic liver cirrhosis: *n* = 51, median MELD 12.1 LTR with history of alcoholic liver cirrhosis: *n* = 61 Alcohol use total group: NS	Self‐report EtOH MeOH CDT hEtG PEth			LTC + LTR, any use			
						71	98	90	95
						LTC + LTR, any use, combination with EtOH and MeOH			
						73	98	91	95
*Biomarker: EtS in urine*									
Stewart and colleagues (2013)	3–7 days	Total: *n* = 120 HCV: *n* = 41; ALD: *n* = 25; HCV + ALD: *n* = 13; NAFLD: *n* = 16; other: *n* = 25 Of which cirrhosis: *n* = 65, median MELD 10 Alcohol use total group: median 16 drinks/wk	Self‐report Clinical assessment CDT PEth	LC‐MS/MS	>25 ng/ml	Cirrhotics + noncirrhotics, 3 days, any use			
						82	86	70	93
						Cirrhotics + noncirrhotics, 7 days, any use			
						73	89	80	85

ALD, alcohol‐related liver disease; AST, aspartate aminotransferase; CDT, carbohydrate deficient transferrin; GGT, gamma glutamyl transferase; EIA, enzyme immunoassay; EtG, ethyl glucuronide; EtOH, ethanol; EtS, ethyl sulfate; HCV, hepatitis C virus; hEtG, hair ethyl glucuronide; LC‐MS/MS, liquid chromatography‐tandem mass spectrometry; LTC, liver transplant candidates; LTR, liver transplant recipients; MCV, mean corpuscular volume; MELD, model for end‐stage liver disease; MeOH; methanol; NA, not assessed; NAFLD, nonalcoholic fatty liver disease; NPV, negative predictive value; NS, not specified; PEth, phosphatidylethanol; PPV, positive predictive value; SE, sensitivity; SP, specificity; uEtG, urinary ethyl glucuronide; uEtS, urinary ethyl sulfate.

**Table 2:** Diagnostic Accuracy of Mid‐Term Biomarkers of Alcohol Use in Patients With Liver Disease

Study	Diagnostic time window	Study population	Reference standard	Method	Cutoff value	Diagnostic accuracy			
						SE	SP	PPV	NPV
*Biomarker: CDT in blood*									
Imbert‐Bismut and colleagues (2009)	NS	Total: *n* = 320 ALD with alcohol use ≥50 g/d past year: *n* = 97, of which cirrhosis: *n* = 38 Nonalcoholic liver disease: *n* = 171, of which NAFLD: *n* = 72; HCV: *n* = 71; HBV: *n* = 28 Healthy volunteers with alcohol use <30 g/d past year: *n* = 52	Self‐report Family report	N‐latex	>2.5%	Cirrhotics + noncirrhotics, ≥30 g/d			
						44	99	96	71
Staufer and colleagues (2011)	NS	Total: *n* = 141 LTC with alcoholic liver cirrhosis: *n* = 105 LTR with history of alcoholic liver cirrhosis: *n* = 36 Alcohol use total group: NS	Self‐report EtOH MeOH uEtG uEtS	HPLC	>2.6%	LTC + LTR, any use			
						25	98.6	63.6	92.9
Gonzalo and colleagues (2012)	15 days	Total: *n* = 110 Alcoholic liver cirrhosis: *n* = 40 Nonalcoholic liver cirrhosis: *n* = 13 Combined cirrhosis: *n* = 8 Noncirrhotic volunteers: *n* = 49 Alcohol use total group: <60 g/d: *n* = 91; ≥60 g/d: *n* = 19	Self‐report Family report EtOH (blood and urine)	CZE	Neg: <1.3% Pos: ≥1.7%	Noncirrhotics, ≥60 g/d			
						86	94		
						Cirrhotics, ≥60 g/d			
						40	83		
						Cirrhotics, criterium 17 ≤ ∆ ≤ 21^a^, ≥60 g/d			
						100	96		
				N‐latex	≥3%	Cirrhotics, ≥60 g/d			
						67	57		
Fagan and colleagues (2014)	2 weeks	Total: *n* = 52 Heavy alcohol drinkers with use ≥50 g/d (females)/≥60 g/d (males) in previous 6 months: *n* = 52 Of which cirrhosis: *n* = 18, CTP‐A: *n* = 10; CTP‐B: *n* = 7; CTP‐C: *n* = 1	Self‐report	HPLC	>1.7%	Heavy alcohol drinkers, ≥50 to 60 g/d			
						50			
Piano et al. (2014)	NS	Total: *n* = 121 LTC with alcoholic liver cirrhosis: *n* = 98 LTR with history of alcoholic liver cirrhosis: *n* = 23 Alcohol use total group: mean 14.1 drinks/wk	Self‐report EtOH (blood, urine) uEtG	HPLC	>2.1%	LTC + LTR, any use			
						29.7	96.4	78.6	75.5
					>2.1%	LTC, any use			
						25	95.5	72.7	72.4
Tamigniau and colleagues (2014)	NS	Total: *n* = 372 Heavy alcohol drinkers with use >210 g/wk: *n* = 243, of which cirrhosis: *n* = 28 Abstinence with cirrhosis: *n* = 44, of which alcoholic: *n* = 21; viral: *n* = 6; other: *n* = 17 Healthy volunteers: *n* = 85	Self‐report Medical files	CZE	≥1.0%	Heavy alcohol drinkers, >210 g/wk			
						79.4	89.4		
					≥1.3%	Heavy alcohol drinkers, >210 g/wk			
						72.0			
					≥1.6%	Heavy alcohol drinkers, >210 g/wk			
						63.4			
						Heavy alcohol drinkers, F3^b^, >210 g/wk			
						48.1			
						Heavy alcohol drinkers, F4^b^, >210 g/wk			
						59.1			
					≥0.73%	Heavy alcohol drinkers, >210 g/wk, combination with TST			
						86.8	94.1		
Andresen‐Streichert and colleagues (2017)	4 weeks	Total: *n* = 112 LTC with alcoholic liver cirrhosis: *n* = 51, median MELD 12.1 LTR with history of alcoholic liver cirrhosis: *n* = 61 Alcohol use total group: NS	Self‐report EtOH MeOH hEtG uEtG PEth	HPLC	>2.6%	LTC + LTR, any use			
						21	100	100	86
*Biomarker: PEth in blood*									
Stewart and colleagues (2014)	30 days	Total: *n* = 222 HCV: *n* = 74; ALD: *n* = 51; NAFLD: *n* = 32; HCV + ALD: *n* = 27; AIH: *n* = 18; cryptogenic: *n* = 10; other: *n* = 20 Of which cirrhosis: *n* = 196, median MELD 10 Alcohol use total group: abstinence: *n* = 94; <4 drinks/d: *n* = 93; ≥4 drinks/d: *n* = 35	Self‐report Clinical assessment hEtG uEtG	LC‐MS/MS	≥8 ng/ml	Cirrhotics + noncirrhotics, any use			
						79	90		
					≥20 ng/ml	Cirrhotics + noncirrhotics, any use			
						73	96		
						Cirrhotics + noncirrhotics, ≥4 drinks/d			
						97	66		
					≥80 ng/ml	Cirrhotics + noncirrhotics, ≥4 drinks/d			
						91	77		
Andresen‐Streichert and colleagues (2017)	1 week	Total: *n* = 112 LTC with alcoholic liver cirrhosis: *n* = 51, median MELD 12.1 LTR with history of alcoholic liver cirrhosis: *n* = 61 Alcohol use total group: NS	Self‐report EtOH MeOH CDT hEtG uEtG	Online SPE‐LC‐MS/ MS in DBS	>20 ng/ml	LTC + LTR, any use			
						100	96	85	100

AIH, auto‐immune hepatitis; ALD, alcohol‐related liver disease; AST, aspartate aminotransferase; BMI, body mass index; CDT, carbohydrate deficient transferrin; CTP, Child‐Turcotte‐Pugh score; CZE, capillary zone electrophoresis; DBS, dried blood spots; FN, false negative; GGT, gamma glutamyl transferase; EtOH, ethanol; HBV, hepatitis B virus; HCV, hepatitis C virus; hEtG, hair ethyl glucuronide; HPLC, high performance liquid chromatography; LC‐MS/MS, liquid chromatography‐tandem mass spectrometry; LTC, liver transplant candidates; LTR, liver transplant recipients; MCV, mean corpuscular volume; MELD, model for end‐stage liver disease; MeOH; methanol; NA, not assessed; NAFLD, nonalcoholic fatty liver disease; NPV, negative predictive value; NS, not specified; PEth, phosphatidylethanol; PPV, positive predictive value; SE, sensitivity; SP, specificity; SPE‐LC‐MS/MS, solid‐phase extraction liquid chromatography‐tandem mass spectrometry; TST, trisialotransferrin; uEtG, urinary ethyl glucuronide; uEtS, urinary ethyl sulfate.

^a^ Delta‐time (∆) is an indicator of the apparent resolution between di‐ and trisialotransferrin peaks, and this parameter was used as a filtering criterium to select electrophoretic profiles that could reliably be interpreted.

^b^ Fibrosis stage assessed by transient elastography (FibroScan) and classified according to the METAVIR scoring system for fibrosis.

**Table 3:** Diagnostic Accuracy of Long‐Term Biomarkers of Alcohol Use in Patients With Liver Disease

Study	Diagnostic time window	Study population	Reference standard	Method	Cutoff value	Diagnostic accuracy			
						SE	SP	PPV	NPV
*Biomarker: ETG in hair*									
Stewart and colleagues (2013)	3 months	Total: *n* = 191 Liver disease (NS): *n* = 191 Of which cirrhosis: *n* = 103 Alcohol use total group: abstinence: *n* = 82;> 0, <28 g/d: *n* = 57; ≥28 g/d: *n* = 52	Self‐report Clinical assessment uEtG PEth	LC‐MS/MS	≥8 pg/mg	Cirrhotics + noncirrhotics, any use			
						58	99		
						Cirrhotics, any use			
						65	98		
					≥8 pg/mg	Cirrhotics + noncirrhotics, ≥28 g/d			
						90	88		
						Cirrhotics, use ≥28			
						100	94		
					≥30 pg/mg	Cirrhotics + noncirrhotics, ≥28 g/d			
						81	93		
Sterneck and colleagues (2014)	3 to 6 months	Total: *n* = 88 LTC with alcoholic liver cirrhosis: *n* = 63, mean MELD 16.65 Nonalcoholic liver cirrhosis, not presenting for LTx, abstinence: *n* = 25 Alcohol use total group: NS	Self‐report EtOH MeOH CDT uEtG	GC/MS	≥7 pg/mg	LTC, >10 g/d, 3 to 6 months			
						76	91	71	94
					≥30 pg/mg	LTC, >60 g/d, 3 months			
						86	98	92	86
						LTC, >60 g/d, 3 to 6 months			
						85	97	85	89
Andresen‐Streichert and colleagues (2017)	3 months	Total: *n* = 112 LTC with alcoholic liver cirrhosis: *n* = 51, median MELD 12.1 LTR with history of alcoholic liver cirrhosis: *n* = 61 Alcohol use total group: NS	Self‐report EtOH MeOH CDT uEtG PEth	LC‐MS/MS	≥7 pg/mg	LTC + LTR together, any use			
						84	92	68	96
Verbeek and colleagues (2018)	3 months	Total: *n* = 101 Alcoholic liver cirrhosis: *n* = 58 Healthy volunteers: *n* = 43 Alcohol use cirrhotic patients: abstinence: *n* = 30;> 0, <60 g/d: *n* = 9; ≥60 g/d: *n* = 19	Self‐report Clinical assessment	GC‐MS/MS	≥7, <30 pg/mg	Cirrhotics, >0, <60 g/d			
						67	66	38	86
					≥30 pg/mg	Cirrhotics, ≥60 g/d			
						100	97	95	100
					≥50 pg/mg	Cirrhotics, use ≥60 g/d			
						100	100	100	100

CDT, carbohydrate‐deficient transferrin; EtG, ethyl glucuronide; EtOH, ethanol; GC‐MS, gas chromatography‐mass spectrometry; GC‐MS/MS, gas chromatography‐tandem mass spectrometry; LC‐MS/MS, liquid chromatography‐tandem mass spectrometry; LTC, liver transplant candidates; LTR, liver transplant recipients; LTx, liver transplantation; MELD, model for end‐stage liver disease; MeOH; methanol; NA, not assessed; NPV, negative predictive value; NS, not specified; PEth, phosphatidylethanol; PPV, positive predictive value; SE, sensitivity; SP, specificity; uEtG, urinary ethyl glucuronide.

#### Short‐Term Biomarkers of Alcohol Use

Four studies assessed the diagnostic accuracy of the short‐term biomarker uEtG in patients with liver disease (Table 1). The assessed diagnostic time windows ranged from 3 to 7 days (Andresen‐Streichert et al., 2017; Stewart et al., 2013a). Most study populations consisted of both liver transplant candidates (LTC) with alcohol‐related liver cirrhosis and liver transplant recipients (LTR) with a history of alcohol‐related liver cirrhosis (Andresen‐Streichert et al., 2017; Piano et al., 2014; Staufer et al., 2011). One study included patients with liver disease with and without cirrhosis (Stewart et al., 2013a). The diagnostic accuracy of uEtG per study can be found in Table 1.

Urinary EtG correlated with the reported amount of alcohol used (*p* < 0.001; Piano et al., 2014; Stewart et al., 2013a) and with uEtS results (*p* < 0.001; Stewart et al., 2013a). Moreover, uEtG outperformed CDT in the prediction of alcohol use (*p* < 0.001; Piano et al., 2014; Staufer et al., 2011). However, SE (71%) of uEtG for any alcohol use (mean alcohol intake of the study population not reported) in the past week was significantly lower in comparison to PEth (100%) in a population consisting of both LTC and LTR (*p* = 0.046; Andresen‐Streichert et al., 2017). In 1 patient, alcohol use was detected by uEtG after 6 confirmed days of abstinence, yet this patient suffered from acute kidney injury (Stewart et al., 2013a).

Diagnostic accuracy of uEtS was assessed in only 1 study on patients with cirrhotic or noncirrhotic liver disease. The SE ranged from 73 to 82% and SP ranged from 86 to 89%, depending on the applied diagnostic time window and cutoff value (Stewart et al., 2013a; Table 1). Results of uEtS correlated with uEtG results (*p* < 0.001) and with the reported amount of alcohol used (*p* < 0.001). In this study, liver disease severity (represented by the presence of cirrhosis and Child‐Turcotte‐Pugh [CTP] score and model for end‐stage liver disease [MELD] score for subjects with cirrhosis) did not significantly affect the correlation between alcohol consumption and biomarker positivity of uEtG and uEtS and neither did age, gender, or ethnicity (all *p* > 0.250; Stewart et al., 2013a).

#### Mid‐Term Biomarkers of Alcohol Use

Seven studies on the diagnostic accuracy of the mid‐term biomarker CDT were included (Table 2). The assessed diagnostic time windows ranged from 2 to 4 weeks (Andresen‐Streichert et al., 2017; Fagan et al., 2014; Gonzalo et al., 2012). Study populations were highly heterogeneous and consisted of both LTC with alcohol‐related liver cirrhosis and LTR with a history of alcohol‐related liver cirrhosis (Andresen‐Streichert et al., 2017; Piano et al., 2014; Staufer et al., 2011), heavy alcohol drinkers with or without cirrhosis (Fagan et al., 2014; Tamigniau et al., 2014), and patients with alcohol‐related and nonalcoholic liver disease with or without cirrhosis (Gonzalo et al., 2012; Imbert‐Bismut et al., 2009). The diagnostic accuracy of CDT per study is listed in Table 2.

CDT correlated with the amount of weekly alcohol used (*p* < 0.01; Piano et al., 2014). Studies on both LTC and LTR reported a lower performance of CDT compared to uEtG for the detection of any amount of alcohol use (*p* < 0.001; Piano et al., 2014; Staufer et al., 2011).

Several studies assessed the influence of liver disease severity on the diagnostic performance of CDT. All of them reported a diminished performance in cirrhotic patients (Fagan et al., 2014; Gonzalo et al., 2012; Piano et al., 2014; Tamigniau et al., 2014). When comparing the diagnostic accuracy for identifying any alcohol use (mean alcohol intake 14.1 drinks/wk), CDT performed worse in LTC versus LTR (SE 25% vs. 60%, SP 96% vs. 100%, PPV 73% vs. 100%, NPV 72% vs. 90%; Piano et al., 2014). For identifying heavy alcohol use (≥60 g/d), CDT performed worse in cirrhotic versus noncirrhotic liver disease patients (SE 40% vs. 86% and SP 83% vs. 94%; Gonzalo et al., 2012), with more false negative results in cirrhotic patients versus noncirrhotic patients (*p* = 0.007; Fagan et al., 2014). Also, cirrhosis was found to be associated with increasing numbers of uninterpretable profiles by using CZE or HPLC because of bridging of di‐ and trisialotransferrin (Gonzalo et al., 2012; Piano et al., 2014) or spectral interference by other biomolecules with similar electrophoretic characteristics (e.g., bilirubin and hemoglobin; Gonzalo et al., 2012). Similar performance in patients with and without cirrhosis only can be established by analyzing profiles with proper separation of di‐ and trisialotransferrin to avoid misinterpretation based on di‐ and trisialotransferrin bridging. One study on cirrhotic patients showed all results were interpretable by using the immunonephelometric N‐latex assay instead of CZE or HPLC, but false‐positive and false‐negative rates were high (SE 67%, SP 57%; Gonzalo et al., 2012). Beside a diminished performance in cirrhotic patients, elevated BMI and female gender were found to reduce diagnostic sensitivity of CDT analyzed by HPLC in a cohort of patients with heavy alcohol consumption (of which 35% were cirrhotic patients; Fagan et al., 2014).

Diagnostic accuracy of PEth, a newer direct mid‐term biomarker of alcohol use, was assessed in 2 included studies. Any past month drinking and any past week drinking were analyzed. Study populations consisted of both cirrhotic‐ and noncirrhotic liver disease patients (Stewart et al., 2014) and both LTC with alcohol‐related liver cirrhosis and LTR with a history of alcohol‐related cirrhosis (Andresen‐Streichert et al., 2017). The diagnostic accuracy of PEth per study can be found in Table 2. In a study population consisting of patients with a variety of liver disease, the relationship between PEth concentration and alcohol use did not depend on liver disease severity (i.e., cirrhotic vs. noncirrhotic patients, *p* = 0.280) and diagnostic performance was not influenced by gender (*p* = 0.210) or age (*p* = 0.438; Stewart et al., 2014).

#### Long‐Term Biomarkers of Alcohol Use

In the 4 included studies on hEtG in patients with liver disease (Table 3), diagnostic accuracy was assessed in liver disease patients with or without cirrhosis (Stewart et al., 2013b), in LTC with alcohol‐related liver cirrhosis (Sterneck et al., 2014), in LTC in combination with LTR (Andresen‐Streichert et al., 2017), and in patients with alcohol‐related liver cirrhosis (Verbeek et al., 2018a). The assessed diagnostic time window was 3 months (analysis proximal 3‐cm hair segment) in all studies and 1 study also determined diagnostic accuracy for past 6‐month drinking (analysis proximal 6‐cm hair segment; Sterneck et al., 2014). Most studies (Andresen‐Streichert et al., 2017; Sterneck et al., 2014; Verbeek et al., 2018a) used hEtG cutoff values previously proposed by the society of hair testing (SoHT; i.e., <7 pg/mg for abstinence; 7–30 pg/mg for moderate alcohol use and ≥30 pg/mg for chronic excessive alcohol use in the previous 3 months; SoHT, 2016). The diagnostic accuracy of hEtG per study is listed in Table 3.

Hair EtG correlated with the average amount of daily alcohol used (*p* = 0.002; Stewart et al., 2013b). Liver disease severity assessed by bilirubin, albumin, international normalized ratio (INR), MELD, and CTP score did not differ between hEtG‐positive LTC and hEtG‐negative LTC (all *p* > 0.223; Sterneck et al., 2014) and liver disease severity assessed by bilirubin, INR, and MELD score did not differ between patients with low and high hEtG levels (all *p* > 0.05; Verbeek et al., 2018a). One study in 191 patients with liver disease reported a better diagnostic accuracy in cirrhotics versus noncirrhotics (*p* < 0.05; Stewart et al., 2013b). For the detection of moderate to heavy alcohol use (≥28 g/d) in the past 3 months for example, both SE and SP increased when comparing patients with and without cirrhosis (SE 100% vs. 90%, SP 94% vs. 88%). This study also reported a modest diminished performance in women versus men (*p* < 0.05). In an analysis limited to women, no significant interaction with hair coloring was found (*p* = 0.269). In 2 studies assessing the influence of renal function on hEtG levels, creatinine levels were not significantly different between hEtG‐positive LTC and hEtG‐negative LTC (*p* = 0.076; Sterneck et al., 2014) and no significant difference between creatinine levels and low or high hEtG levels was found (*p* > 0.05; Verbeek et al., 2018a).

## Discussion

Multiple biomarkers (uEtG, PEth, hEtG) show promising diagnostic accuracies in patients with liver disease. Cirrhosis can theoretically impair the diagnostic accuracy of alcohol biomarkers by altered hepatic biomarker formation and altered growth of hair and nail (Cederbaum, 2012). Furthermore, patients with liver disease have a higher prevalence of kidney dysfunction and use of diuretics that might lead to a changed renal excretion of biomarkers (Cederbaum, 2012). However, only a limited number of studies in patients with liver disease have been performed so far and some issues on the quality and applicability of these studies were raised. The main limitation of all studies is the lack of an absolute gold standard, which is, however, the incentive of these studies. Second, most of the reported diagnostic accuracies were based on analyses in study populations consisting of both cirrhotic and noncirrhotic patients, without assessing the diagnostic accuracy for solely cirrhotic patients and without sufficiently assessing possible confounding factors that are often present in patients with cirrhosis.

UEtG and uEtS were found to be highly specific to detect any amount of alcohol consumption in the past days (Andresen‐Streichert et al., 2017; Piano et al., 2014; Staufer et al., 2011; Stewart et al., 2013a). Their sensitivity seems to be somewhat lower, and thus, light or even moderate drinking could be missed (Stewart et al., 2013a). False‐negative uEtG results may result from urine dilution by using diuretics (Goll et al., 2002) and from bacterial degradation in urine (e.g., urinary tract infections; Helander and Dahl, 2005). The use of diuretics, common in cirrhotic patients, was not reported in any of the studies. However, most studies determined urinary creatinine values to account for intentional dilution of the urine samples (Piano et al., 2014; Staufer et al., 2011; Stewart et al., 2013a). In contrast, severe renal dysfunction (Wurst et al., 2004), unintentional exposure to small amounts of alcohol (e.g., mouthwash solutions, baker’s yeast, medication; Reisfield et al., 2011; Thierauf et al., 2010) and postcollection synthesis of uEtG from alcohol by bacteria (Helander et al., 2007) may cause false‐positive results.

CDT is the most studied and most widely used biomarker to assess excessive alcohol use in the past weeks (Andresen‐Streichert et al., 2017; Fagan et al., 2014; Gonzalo et al., 2012; Imbert‐Bismut et al., 2009; Piano et al., 2014; Staufer et al., 2011; Tamigniau et al., 2014). Despite its rather high specificity, CDT shows poor sensitivity, in particular in patients with cirrhosis (Fagan et al., 2014; Gonzalo et al., 2012; Imbert‐Bismut et al., 2009; Piano et al., 2014; Staufer et al., 2011; Tamigniau et al., 2014). Cirrhosis may lead to poor chromatographic and electrophoretic separation of transferrin isoforms leading to di‐ and tri‐sialotransferrin bridging (Gonzalo et al., 2012; Piano et al., 2014; Verbeek et al., 2018b), which impairs the interpretation of CDT as a marker of (heavy) alcohol consumption in these patients. Therefore, in our opinion, CDT is only of limited value in patients with liver disease and the clinician should be aware of the applied analysis method and the related cutoff levels to categorize patients as excessive alcohol users. In contrast, PEth shows high sensitivity and specificity to detect any or excessive alcohol use in the past weeks irrespective of the presence and stage of liver disease, with a detection window comparable with CDT (i.e., a few weeks; Stewart et al., 2014). However, extra validation studies and in particular more laboratories with the expertise to perform PEth analysis are needed.

HEtG is highly sensitive and specific for the detection of chronic excessive alcohol use in the past months, even in the presence of cirrhosis (Sterneck et al., 2014; Stewart et al., 2013b; Verbeek et al., 2018a) and regardless of the stage of liver dysfunction (Sterneck et al., 2014; Verbeek et al., 2018a). However, hEtG does not perform well in differentiating abstinence from ongoing light to moderate alcohol use (Andresen‐Streichert et al., 2017; Sterneck et al., 2014; Stewart et al., 2013b; Verbeek et al., 2018a). In our previous study, we found that a significant proportion of our abstinent patients with cirrhosis and prior alcohol abuse still had increased hEtG levels, in contrast to abstinent healthy volunteers who all had normal levels of <7 pg/mg (Crunelle et al., 2016, 2017). This finding suggests that for patients with cirrhosis and prior alcohol abuse other cutoff values than those proposed by the SoHT (2016) should be used. Remaining hEtG and differences in hair growth in patients with cirrhosis might be an explanation (EASL, 2018). There are significant differences in the proportion anagen (active growing) and telogen (resting) hair, depending on patients’ health state (Pragst and Balikova, 2006). Consequently, hair age heterogeneity may result in alterations of alcohol biomarker distribution along the hair. Serial or segmental hair analyses could give more insight in the changed drinking patterns, but these are labor‐ and time‐intensive procedures. Importantly, hEtG levels should always be interpreted with caution in patients with severe renal impairment (Fosen et al., 2016; Hoiseth et al., 2013) and cosmetic hair treatment (i.e., bleaching, perming, coloring; Crunelle et al., 2015; Kerekes and Yegles, 2013) because false‐positive and false‐negative interpretation, respectively, may occur.

## Conclusion and Future Perspectives

Alcohol use biomarkers based on EtG are currently the most validated ones and are already applied in clinical care. Measurements of EtG in urine and scalp hair complement each other regarding diagnostic time window. Knowledge of their capacities and limitations is important to avoid misclassification of patients with ALD regarding their abstinence or alcohol use, especially in the context of LTx. Therefore, additional validation studies of in patients with liver disease, focusing on the determination of cutoff levels and identification of confounding factors, could further establish the place of these biomarkers in clinical practice. We foresee that the highly promising mid‐term direct marker PEth will gain importance over CDT in the near future. Analysis of EtG in nails might offer advantages as a long‐term alcohol biomarker in case scalp hair is not sufficiently available (Cappelle et al., 2017). However, this application is in its experimental phase and no studies on EtG in nails of patients with cirrhosis are performed so far. Future studies should assess the diagnostic accuracy of alcohol biomarkers for a specific quantity, range, or pattern of alcohol use during a specific diagnostic time window. Studies analyzing the kinetics of positive alcohol use biomarkers in patients with liver disease could provide valuable information to assess half‐lives and determining detection windows and cutoff values. Ideally, alcohol use should be monitored prospectively or at least with the Timeline Followback method (Sobell et al., 2001). Only patients with a defined stage of liver disease should be included, taking into account possible confounding factors. These studies may bring us a step closer to the development of a gold alcohol biomarker(‐set) that is both perfectly sensitive and specific for any amount of alcohol intake, correlates with the consumed amount of alcohol, has a large diagnostic time window, is not subject to intentional manipulation, and is not confounded by other factors.

## Funding

The study was supported by the Maag Lever Darm Stichting (Dutch Digestive Foundation)—Grant number D 18‐19.

## Conflict of interest

The authors declare no conflict of interest with this study.

## Supporting information


**Supinfo S1.** Supplement 1: Quality assessment.
**Fig. S1.** Summary of the quality assessment of the included studies using the adapted QUADAS‐2.
