# Supplementary material for: Diagnostic Accuracy of Biomarkers of Alcohol Use in Patients With Liver Disease: A Systematic Review
Source: Alcohol Clin Exp Res. 2020 Dec 25;45(1):25–37. doi: 10.1111/acer.14512 (PMC7898850; doi:10.1111/acer.14512)
Supplement: Supplementary file 1 — Supinfo S1. Supplement 1: Quality assessment. Fig. S1. Summary of the quality assessment of the included studies using the adapted QUADAS‐2. [file ACER-45-25-s001.docx]

**Supplemental documentation**

**Systematic review: Diagnostic accuracy of biomarkers of alcohol use in patients with liver disease**

Janique Arnts, MD^1*^ Benedict TK Vanlerberghe, MD^1*^, Sylvia Roozen, PhD^2^, Cleo L Crunelle, PhD ^3,8^, Ad AM Masclee, MD, PhD^1,4^ Steven WM Olde-Damink, MD, PhD^4,5,6^, Ron MA Heeren, PhD^7^, Alexander van Nuijs, PhD^8^, Hugo Neels, PhD^8^,

Frederik Nevens, MD, PhD ^9^, Jef Verbeek, MD, PhD^9^

^1^Division of Gastroenterology & Hepatology, Department of Internal Medicine, Maastricht University Medical Center, Maastricht, The Netherlands

^2^Governor Kremers Centre-Maastricht University Medical Centre, Maastricht, The Netherlands

^3^Vrije Universiteit Brussel (VUB), Universitair Ziekenhuis Brussel (UZ Brussel), Department of Psychiatry, Brussels, Belgium

^4^NUTRIM School of Nutrition and Translational Research in Metabolism, Maastricht University, Maastricht, The Netherlands

^5^Department of Surgery, Maastricht University Medical Center, Maastricht, The Netherlands

^6^Department of General, Visceral and Transplantation Surgery, RWTH University Hospital Aachen, Aachen, Germany

^7^Maastricht MultiModal Molecular Imaging (M4I) Institute, Division of Imaging Mass Spectrometry, Maastricht University, Maastricht, The Netherlands.

^8^Toxicological Center, University of Antwerp, Antwerp, Belgium

^9^Department of Gastroenterology & Hepatology, University Hospitals KU Leuven,

**Supplement 1: Quality assessment**

The methodological quality of included studies was evaluated according to the quality assessment of diagnostic accuracy studies 2 (QUADAS-2) checklist(Whiting et al., 2011). This scale consists of four main domains: 1) patient selection; 2) index test (i.e. alcohol biomarker); 3) reference standard and 4) flow and timing. All of these domains were assessed for risk of bias and the first three domains also were assessed for applicability concerns, rated as: 1) low; 2) high or 3) unclear (Supplementary table 1). Review specific tailoring of the QUADAS-2 was performed and two of the researchers (J.A and S.R.) independently assessed all included articles for quality. Differences in interpretation were resolved by discussion. Supplementary figure 1 summarizes the risk of bias and applicability concerns of all included studies using the adapted QUADAS-2 quality assessment tool (Supplementary table 1).

***Risk of bias***

*Patient selection*

In six studies(Gonzalo et al., 2012, Fagan et al., 2014, Stewart et al., 2014, Tamigniau et al., 2014, Andresen-Streichert et al., 2017, Verbeek et al., 2018) it was unclear if the patient sampling could have introduced bias. In these studies, it was not well reported if a random or consecutive sampling method was used instead of for example using a non-probability convenience sampling method. All studies avoided a case-control design and appropriate exclusion criteria were used.

*Index test*

It is unlikely that the applied index tests (i.e. methods for alcohol biomarker analysis) could have introduced bias. All studies reported well on the method of analysis, validated methods were used and cut-off values corresponding with negative and positive alcohol biomarker test results were specified before testing.

*Reference standard*

All studies relied on self-reported alcohol intake, which can be unreliable because patients may underestimate their actual alcohol use or cannot recall the exact amount of alcohol used in the past period. Moreover, it is recommended to use the timeline follow-back (TLFB) method(EASL, 2018), but several studies did not specify which questionnaire was used(Gonzalo et al., 2012, Staufer et al., 2011, Sterneck et al., 2014, Tamigniau et al., 2014) or used other or self-designed alcohol questionnaires(Andresen-Streichert et al., 2017, Verbeek et al., 2018, Fagan et al., 2014, Imbert-Bismut et al., 2009, Piano et al., 2014). Several studies used blood alcohol levels as a confirmatory method(Gonzalo et al., 2012, Piano et al., 2014, Andresen-Streichert et al., 2017, Staufer et al., 2011), but this will not be fully diagnostic due to its shorter diagnostic time window (i.e. 4 – 12 h(EASL, 2018)). Several studies used other alcohol biomarkers to make self-reports more reliable(Staufer et al., 2011, Piano et al., 2014, Andresen-Streichert et al., 2017, Stewart et al., 2013a, Stewart et al., 2013b, Stewart et al., 2014, Sterneck et al., 2014, Verbeek et al., 2018) but this still yields a risk of bias. Taken together, due to the lack of an absolute gold standard to assess any amount of alcohol use over a past period of more than one day, the risk of bias with respect to the used reference standard is a concern for all the included studies.

*Flow and timing*

All patients included in the individual studies received the same reference standard and there was no or minimal loss to follow up. It is worth noting that in several studies on CDT all patients were analysed but some samples were uninterpretable due to poor electrophoretic or chromatographic profiles and in these cases, patients were excluded from diagnostic accuracy assessments(Tamigniau et al., 2014, Piano et al., 2014, Gonzalo et al., 2012).

***Applicability concerns***

*Patient selection*

Most studies included both cirrhotic and non-cirrhotic patients and analyzed whether or not alcohol biomarker results were influenced by the presence and severity of liver disease (i.e. studies reported a diagnostic accuracy for solely cirrhotic patients or a p-value for the influence of liver disease severity on alcohol biomarker concentration was given). However, in three studies diagnostic accuracy was assessed taking together liver transplant candidates (LTC) and liver transplant recipients (LTR) without assessing the influence of cirrhosis on the diagnostic accuracy of the alcohol biomarkers of interest(Staufer et al., 2011, Piano et al., 2014, Andresen-Streichert et al., 2017).

*Index test*

Two studies(Andresen-Streichert et al., 2017, Sterneck et al., 2014) did not report the (mean) amount of alcohol consumption of the study population, while the diagnostic accuracy may be influenced by the specific amount of alcohol used in the study population. The higher the amount of alcohol used, the higher the sensitivity of a biomarker for any alcohol use will be. Moreover, four studies did not specify the diagnostic time window in their study set-up, so it remains unclear to which diagnostic time window the reported diagnostic values are related(Piano et al., 2014, Tamigniau et al., 2014, Staufer et al., 2011, Imbert-Bismut et al., 2009).

*Reference standard*

All studies used a reference standard that detected a specific amount or range of alcohol use, which matches the target condition of our current review question.

**Supplementary Figure 1:** Summary of the quality assessment of the included studies using the adapted QUADAS-2. A. Quality assessment results of individual studies. B. Summary graph of quality of included studies.

*See following page*

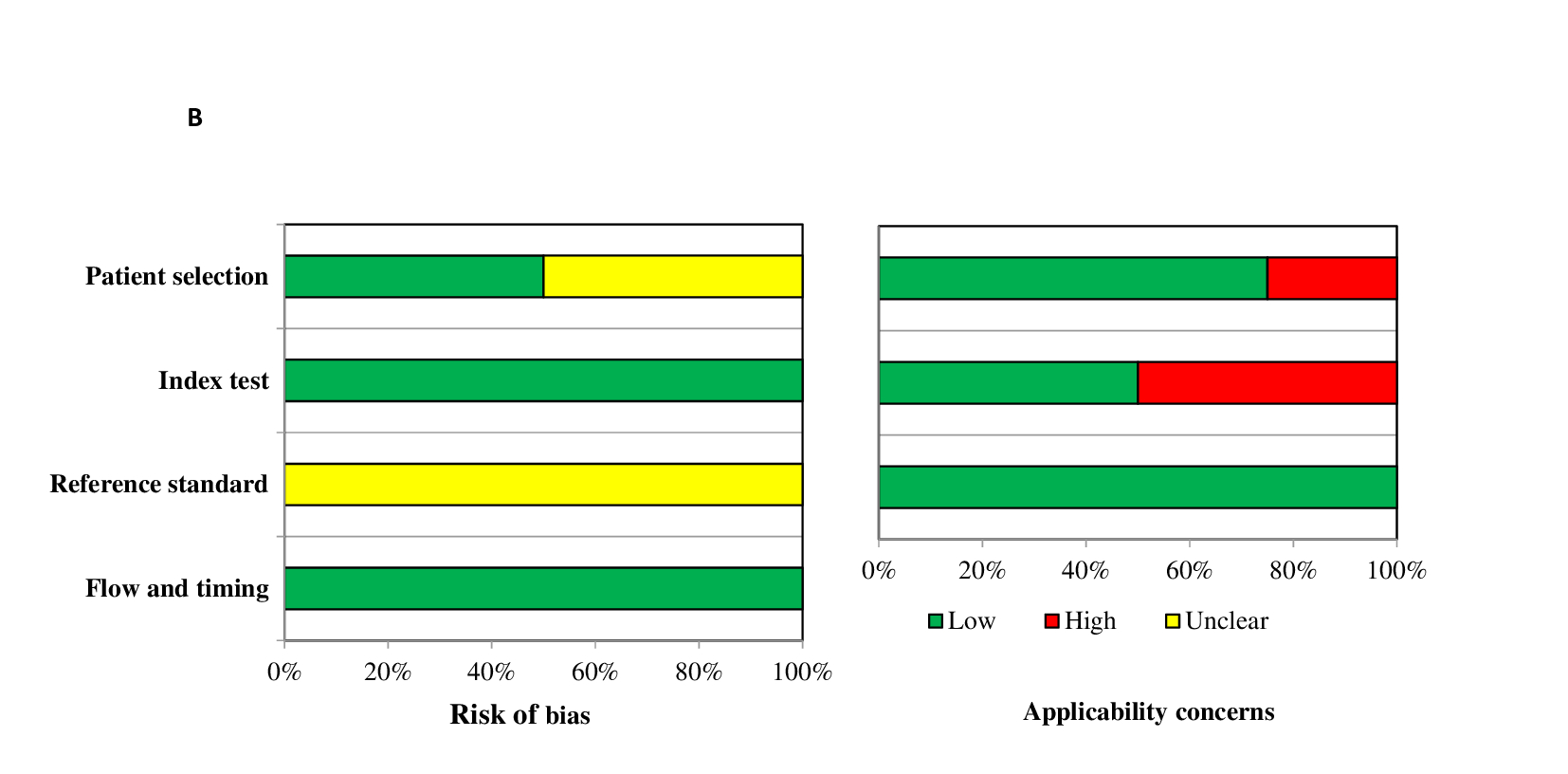


**REFERENCES**

ANDRESEN-STREICHERT, H., BERES, Y., WEINMANN, W., SCHROCK, A., MULLER, A., SKOPP, G., PISCHKE, S., VETTORAZZI, E., LOHSE, A., NASHAN, B. & STERNECK, M. 2017. Improved detection of alcohol consumption using the novel marker phosphatidylethanol in the transplant setting: results of a prospective study. *Transpl Int,* 30**,** 611-620.

EASL 2018. Clinical Practice Guidelines: Management of alcohol-related liver disease. European Association for the Study of the Liver.

FAGAN, K. J., IRVINE, K. M., MCWHINNEY, B. C., FLETCHER, L. M., HORSFALL, L. U., JOHNSON, L., O'ROURKE, P., MARTIN, J., SCOTT, I., PRETORIUS, C. J., UNGERER, J. P. & POWELL, E. E. 2014. Diagnostic sensitivity of carbohydrate deficient transferrin in heavy drinkers. *BMC Gastroenterol,* 14**,** 97.

GONZALO, P., PECQUET, M., BON, C., GONZALO, S., RADENNE, S., AUGUSTIN-NORMAND, C. & SOUQUET, J. C. 2012. Clinical performance of the carbohydrate-deficient transferrin (CDT) assay by the Sebia Capillarys2 system in case of cirrhosis. Interest of the Bio-Rad %CDT by HPLC test and Siemens N-Latex CDT kit as putative confirmatory methods. *Clin Chim Acta,* 413**,** 712-8.

IMBERT-BISMUT, F., NAVEAU, S., MORRA, R., MUNTEANU, M., RATZIU, V., ABELLA, A., MESSOUS, D., THABUT, D., BENHAMOU, Y. & POYNARD, T. 2009. The diagnostic value of combining carbohydrate-deficient transferrin, fibrosis, and steatosis biomarkers for the prediction of excessive alcohol consumption. *Eur J Gastroenterol Hepatol,* 21**,** 18-27.

PIANO, S., MARCHIORO, L., GOLA, E., ROSI, S., MORANDO, F., CAVALLIN, M., STICCA, A., FASOLATO, S., FORZA, G., CHIARA FRIGO, A., PLEBANI, M., ZANUS, G., CILLO, U., GATTA, A. & ANGELI, P. 2014. Assessment of alcohol consumption in liver transplant candidates and recipients: the best combination of the tools available. *Liver Transpl,* 20**,** 815-22.

STAUFER, K., ANDRESEN, H., VETTORAZZI, E., TOBIAS, N., NASHAN, B. & STERNECK, M. 2011. Urinary ethyl glucuronide as a novel screening tool in patients pre- and post-liver transplantation improves detection of alcohol consumption. *Hepatology,* 54**,** 1640-9.

STERNECK, M., YEGLES, M., ROTHKIRCH VON, G., STAUFER, K., VETTORAZZI, E., SCHULZ, K. H., TOBIAS, N., GRAESER, C., FISCHER, L., NASHAN, B. & ANDRESEN-STREICHERT, H. 2014. Determination of ethyl glucuronide in hair improves evaluation of long-term alcohol abstention in liver transplant candidates. *Liver Int,* 34**,** 469-76.

STEWART, S. H., KOCH, D. G., BURGESS, D. M., WILLNER, I. R. & REUBEN, A. 2013a. Sensitivity and specificity of urinary ethyl glucuronide and ethyl sulfate in liver disease patients. *Alcohol Clin Exp Res,* 37**,** 150-5.

STEWART, S. H., KOCH, D. G., WILLNER, I. R., ANTON, R. F. & REUBEN, A. 2014. Validation of blood phosphatidylethanol as an alcohol consumption biomarker in patients with chronic liver disease. *Alcohol Clin Exp Res,* 38**,** 1706-11.

STEWART, S. H., KOCH, D. G., WILLNER, I. R., RANDALL, P. K. & REUBEN, A. 2013b. Hair ethyl glucuronide is highly sensitive and specific for detecting moderate-to-heavy drinking in patients with liver disease. *Alcohol Alcohol,* 48**,** 83-7.

TAMIGNIAU, A., WALLEMACQ, P. & MAISIN, D. 2014. Could trisialotransferrin be used as an additional biomarker to CDT in order to improve detection of chronic excessive alcohol intake? *Clin Biochem,* 47**,** 1203-8.

VERBEEK, J., CRUNELLE, C. L., LEURQUIN-STERK, G., MICHIELSEN, P. P., DE DONCKER, M., MONBALIU, D., PIRENNE, J., ROSKAMS, T., VAN DER MERWE, S., CASSIMAN, D., NEELS, H. & NEVENS, F. 2018. Ethyl Glucuronide in Hair Is an Accurate Biomarker of Chronic Excessive Alcohol Use in Patients With Alcoholic Cirrhosis. *Clin Gastroenterol Hepatol,* 16**,** 454-456.

WHITING, P. F., RUTJES, A. W., WESTWOOD, M. E., MALLETT, S., DEEKS, J. J., REITSMA, J. B., LEEFLANG, M. M., STERNE, J. A., BOSSUYT, P. M. & GROUP, Q.-. 2011. QUADAS-2: a revised tool for the quality assessment of diagnostic accuracy studies. *Ann Intern Med,* 155**,** 529-36.

**Supplementary table 1:** Prisma Checklist

| **Section/topic** | **#** | **Checklist item** | **Reported on page #** |
| --- | --- | --- | --- |
| **TITLE** | | |  |
| Title | 1 | Identify the report as a systematic review, meta-analysis, or both. | 1 |
| **ABSTRACT** | | |  |
| Structured summary | 2 | Provide a structured summary including, as applicable: background; objectives; data sources; study eligibility criteria, participants, and interventions; study appraisal and synthesis methods; results; limitations; conclusions and implications of key findings; systematic review registration number. | 3 |
| **INTRODUCTION** | | |  |
| Rationale | 3 | Describe the rationale for the review in the context of what is already known. | 5 - 7 |
| Objectives | 4 | Provide an explicit statement of questions being addressed with reference to participants, interventions, comparisons, outcomes, and study design (PICOS). | 5 - 7 |
| **METHODS** | | |  |
| Protocol and registration | 5 | Indicate if a review protocol exists, if and where it can be accessed (e.g., Web address), and, if available, provide registration information including registration number. | / |
| Eligibility criteria | 6 | Specify study characteristics (e.g., PICOS, length of follow-up) and report characteristics (e.g., years considered, language, publication status) used as criteria for eligibility, giving rationale. | 8-9 |
| Information sources | 7 | Describe all information sources (e.g., databases with dates of coverage, contact with study authors to identify additional studies) in the search and date last searched. | 8 |
| Search | 8 | Present full electronic search strategy for at least one database, including any limits used, such that it could be repeated. | 8 |
| Study selection | 9 | State the process for selecting studies (i.e., screening, eligibility, included in systematic review, and, if applicable, included in the meta-analysis). | 8-10 |
| Data collection process | 10 | Describe method of data extraction from reports (e.g., piloted forms, independently, in duplicate) and any processes for obtaining and confirming data from investigators. | 8-10 |
| Data items | 11 | List and define all variables for which data were sought (e.g., PICOS, funding sources) and any assumptions and simplifications made. | 8-10 |
| Risk of bias in individual studies | 12 | Describe methods used for assessing risk of bias of individual studies (including specification of whether this was done at the study or outcome level), and how this information is to be used in any data synthesis. | SD p2-4 |
| Summary measures | 13 | State the principal summary measures (e.g., risk ratio, difference in means). | 8-9 |
| Synthesis of results | 14 | Describe the methods of handling data and combining results of studies, if done, including measures of consistency (e.g., I^2^) for each meta-analysis. | 10 |

| **Section/topic** | **#** | **Checklist item** | **Reported on page #** |
| --- | --- | --- | --- |
| Risk of bias across studies | 15 | Specify any assessment of risk of bias that may affect the cumulative evidence (e.g., publication bias, selective reporting within studies). | / |
| Additional analyses | 16 | Describe methods of additional analyses (e.g., sensitivity or subgroup analyses, meta-regression), if done, indicating which were pre-specified. | / |
| **RESULTS** | | |  |
| Study selection | 17 | Give numbers of studies screened, assessed for eligibility, and included in the review, with reasons for exclusions at each stage, ideally with a flow diagram. | 9 |
| Study characteristics | 18 | For each study, present characteristics for which data were extracted (e.g., study size, PICOS, follow-up period) and provide the citations. | SD p2-4 |
| Risk of bias within studies | 19 | Present data on risk of bias of each study and, if available, any outcome level assessment (see item 12). | SD p2-4 |
| Results of individual studies | 20 | For all outcomes considered (benefits or harms), present, for each study: (a) simple summary data for each intervention group (b) effect estimates and confidence intervals, ideally with a forest plot. | 11-15 |
| Synthesis of results | 21 | Present results of each meta-analysis done, including confidence intervals and measures of consistency. | / |
| Risk of bias across studies | 22 | Present results of any assessment of risk of bias across studies (see Item 15). | / |
| Additional analysis | 23 | Give results of additional analyses, if done (e.g., sensitivity or subgroup analyses, meta-regression [see Item 16]). | / |
| **DISCUSSION** | | |  |
| Summary of evidence | 24 | Summarize the main findings including the strength of evidence for each main outcome; consider their relevance to key groups (e.g., healthcare providers, users, and policy makers). | 19 |
| Limitations | 25 | Discuss limitations at study and outcome level (e.g., risk of bias), and at review-level (e.g., incomplete retrieval of identified research, reporting bias). | 16-18 |
| Conclusions | 26 | Provide a general interpretation of the results in the context of other evidence, and implications for future research. | 19 |
| **FUNDING** | | |  |
| Funding | 27 | Describe sources of funding for the systematic review and other support (e.g., supply of data); role of funders for the systematic review. | 2 |

*From:*  Moher D, Liberati A, Tetzlaff J, Altman DG, The PRISMA Group (2009). Preferred Reporting Items for Systematic Reviews and Meta-Analyses: The PRISMA Statement. PLoS Med 6(7): e1000097. doi:10.1371/journal.pmed1000097

For more information, visit: **www.prisma-statement.org**.
